# Evaluation of the effectiveness of an online transition planning program for adolescents on the autism spectrum: trial protocol

**DOI:** 10.1186/s13034-016-0137-0

**Published:** 2016-12-28

**Authors:** Megan Hatfield, Marita Falkmer, Torbjorn Falkmer, Marina Ciccarelli

**Affiliations:** 1 School of Occupational Therapy and Social Work, Curtin University, Perth, Australia; 2 Cooperative Research Centre for Living with Autism (Autism CRC), Long Pocket, Brisbane, QLD Australia; 3 School of Occupational Therapy, La Trobe University, Melbourne, Australia; 4 Department of Medicine and Health Sciences (IHM), Linköping University and Pain and Rehabilitation Centre, Linköping, Sweden; 5 School of Education and Communication, Institution of Disability Research, Jönköping University, Jönköping, Sweden

**Keywords:** Asperger’s syndrome, Autism spectrum disorder, Employment, High school, Post-secondary education, Self-determination theory

## Abstract

**Background:**

The transition from high school to post-secondary education and work is difficult for adolescents on the autism spectrum. Transition planning can be an effective way of supporting adolescents on the autism spectrum to prepare for leaving school and to succeed in obtaining employment; however, there is a need for an autism-specific transition planning program with proven effectiveness. This paper describes a trial protocol for evaluating the Better OutcOmes & Successful Transitions for Autism (BOOST-A™); an online interactive program that empowers adolescents on the autism spectrum to plan their transition from school to further study, training, or employment.

**Methods:**

The trial will involve adolescents on the autism spectrum in high school and their parents, who will be alternately assigned to a control group (regular practice) or an intervention group (using the BOOST-A™). The BOOST-A™ was developed using the PRECEDE-PROCEED model, and is based on the self-determination model, and the strengths- and technology-based approaches. It involves participants completing a series of online modules. The primary outcome will be self-determination, because high self-determination has been linked to successful transition to employment among adolescents on the autism spectrum. Secondary outcomes will include domain-specific self-determination, career planning and exploration, quality of life, and environmental support. Data will be obtained from questionnaires completed by the adolescent on the autism spectrum and their parent/s. Data collection will take place at baseline (Time point 1) and 12 months later (Time point 2).

**Discussion and conclusions:**

This trial will provide evidence of the effectiveness of the BOOST-A™ to assist adolescents on the autism spectrum to successfully transition from school.

*Trial registration* #ACTRN12615000119594

## Background

People on the autism spectrum experience difficulties with socialization and communication, as well as restricted interests and repetitive behaviours [1]. The term ‘people on the autism spectrum’ is the one of the preferred terms by members of the autism community [2] and describes people with a diagnosis of autism spectrum disorder, as defined by the Diagnostic and Statistical Manual of Mental Disorders, fifth edition (DSM-5) [1]. This includes individuals with Asperger’s syndrome and pervasive developmental disorder—not otherwise specified, as previously delineated in the DSM-4 [3]. The transition from school to post-school activities is difficult for adolescents on the autism spectrum [4, 5], who are significantly less likely to attend post-secondary education and training than young people with other disabilities [6]. People on the spectrum who have an intellectual ability within or above the average range have difficulty securing employment; only 16% in Australia have full-time employment after leaving school and 33% work part-time [7]. In addition, adolescents on the autism spectrum are three times less likely to participate in vocational activities compared to their peers on the autism spectrum who also have an intellectual disability (ID) [8].

A lack of transition planning can contribute to poor post-school outcomes for adolescents on the autism spectrum [6]. Transition planning involves the setting of personal goals to prepare the adolescent for leaving high school. Transition planning has been linked to improved self-determination, increased rates of employment, improved success in post-secondary education, and higher community participation among adolescents with disability [9]. Unfortunately, current transition planning practices have resulted in inferior outcomes for adolescents on the autism spectrum when compared to adolescents with other disabilities [10]. Only 23% of adolescents with autism are involved in transition planning [11]; and even when they are involved, they are less likely to be active participants in the process. Fewer parents of adolescents with autism perceive the transition planning process as useful [11], and have reported that they want to be more involved in the process [10, 12]. Currently, schools tend to focus on the academic performance of adolescents on the autism spectrum who do not have an ID, rather than engaging them in comprehensive transition planning [13]. In addition, autism-specific challenges are often not taken into consideration. These include difficulties conceptualizing hypothetical future events, managing anxiety, and communicating their preferences to others [13]. Therefore, there is a need for a more tailored transition planning program for students with autism.

Further to this, there is a need for a transition planning program that has proven effectiveness [14]. Current generic transition planning programs have little empirical evidence to prove their efficacy [15] and many focus on limited aspects of transition planning [16–18]; failing to provide an overall guide for adolescents and their parents on how to navigate the entire transition planning process. There is a need for an accessible and tailored transition planning program for adolescents on the autism spectrum that is proven to be effective in improving their self-determination.

This paper describes a trial protocol for the development and evaluation of a transition planning program called the Better OutcOmes & Successful Transitions for Autism (BOOST-A™). The BOOST-A™ aims to target the specific needs of adolescents on the autism spectrum, to empower them to plan their transition from school to further study, training or paid/unpaid work. This trial follows principles of the SPIRIT guidelines for protocols that support high-quality conduct and reporting of clinical trials [19].

### Objectives of the trial

The hypothesis for the trial is that the BOOST-A™ will improve self-determination in adolescents on the autism spectrum transitioning to post-school life. The objectives of the trial are to:

1. determine the effectiveness of the BOOST-A™ in improving self-determination in adolescents on the autism spectrum; and
2. determine the effectiveness of the BOOST-A™ in improving quality of life; access to environmental supports; career planning and exploration; and vocational exploration among adolescents on the autism spectrum.

## Methods

### PRECEDE-PROCEED model

The PRECEDE-PROCEED model [20] was used to guide the development and evaluation of the BOOST-A™. The model provides a stepwise guide to developing evidence-based interventions that meet the needs of the target group [21]. The model has been used to develop previous health interventions [22, 23]. The PRECEDE component guides the development of an intervention through the application of available research and an appropriate theoretical framework [20], and was used in the development of the BOOST-A™. The PROCEED component provides guidance on trialing and evaluating an intervention, and was used to structure the trial of the BOOST-A™.

### Theoretical frameworks

The BOOST-A™ was based on three main theoretical frameworks: the self-determination model, a strengths-based approach, and a technology-based approach.

#### Self-determination model

Self-determination is an individual’s ability to direct their own life; that is, to make choices about the pathway they will take without feeling they have to rely heavily on others [24]. Self-determined people are goal-orientated, have strong problem-solving abilities, and know their strengths and weaknesses. The environment plays a pivotal role in the development of a young person’s self-determination, with the greatest environmental influences being their family, school, and the wider community [25]. Self-determination is influenced by an individual’s sense of autonomy, competence and relatedness; all of which impact on intrinsic motivation [26]. Self-determination can be fostered by incorporating four key facets: promotion of self-knowledge; consistent support between family, school and professionals; opportunities to take risks; and supporting reflective practice [27].

Self-determination has particular importance for people with a disability, because it is a predictor of successful transition into an autonomous adult life, and is crucial to living an empowered life [25, 27–29]. People with developmental disabilities who feel a greater sense of control in their lives are more likely to be employed in the regular workforce [30]. Therefore, the model of self-determination was used to underpin the development of the BOOST-A™.

#### Strengths-based approach

A strengths-based approach advocates focusing on the individual’s strengths, as opposed to the focus on deficits that is often associated with the medical model [31]. The strengths-based approach was developed in the 1980s to challenge the paradigm that disability is a weakness and a fundamental flaw in the individual, and that individuals were to blame for their difficulties [32]. In contrast, a strengths-based approach views the individual as an asset to society and focuses on how the community can support them to leverage their talents, rather than on how the individual can change to meet society’s expectations. The strengths-based approach is being increasingly utilized by many health professionals [33], as well as in the career development arena [34]. In addition, families with children on the autism spectrum who focused on their child’s strengths had a more positive view of disability and described their child as being more resilient [35]. Thus, the strengths-based approach was used in developing the BOOST-A™.

#### Technology-based approach

Technology-based interventions for individuals on the autism spectrum are growing in popularity [36]. A meta-analysis of technology-based training for people with autism supported the effectiveness of these interventions and advocated their use with this population [37]. Furthermore, parents and students with developmental disabilities who used technology in transition planning were significantly more satisfied with the outcomes of the planning process and experienced increased self-determination [16]. The use of technology-based interventions could be particularly relevant for people on the autism spectrum, as they often have an interest in, and aptitude for, technology [38]. An online program also has the potential to increase accessibility to the transition planning process, especially for adolescents and their families living in regional or remote areas. Therefore, a technology-based approach was used for the development of the BOOST-A™.

### Needs assessment

As recommended by the PRECEDE model, a needs assessment was completed to determine the priority areas in transition planning for adolescents on the autism spectrum [20]. The needs assessment encompassed a range of information sources and included both quantitative and qualitative data, as recommended in the literature [39]. The needs assessment involved two phases: (i) a survey of the adolescents on the autism spectrum, their parents and the professionals who work with them; and (ii) interviews with the parents and professionals. In addition, a systematic review was completed to appraise career planning tools for use with individuals on the autism spectrum [40], and a comprehensive literature review was conducted to identify current best-practice in transition planning.

**Fig. 1:**
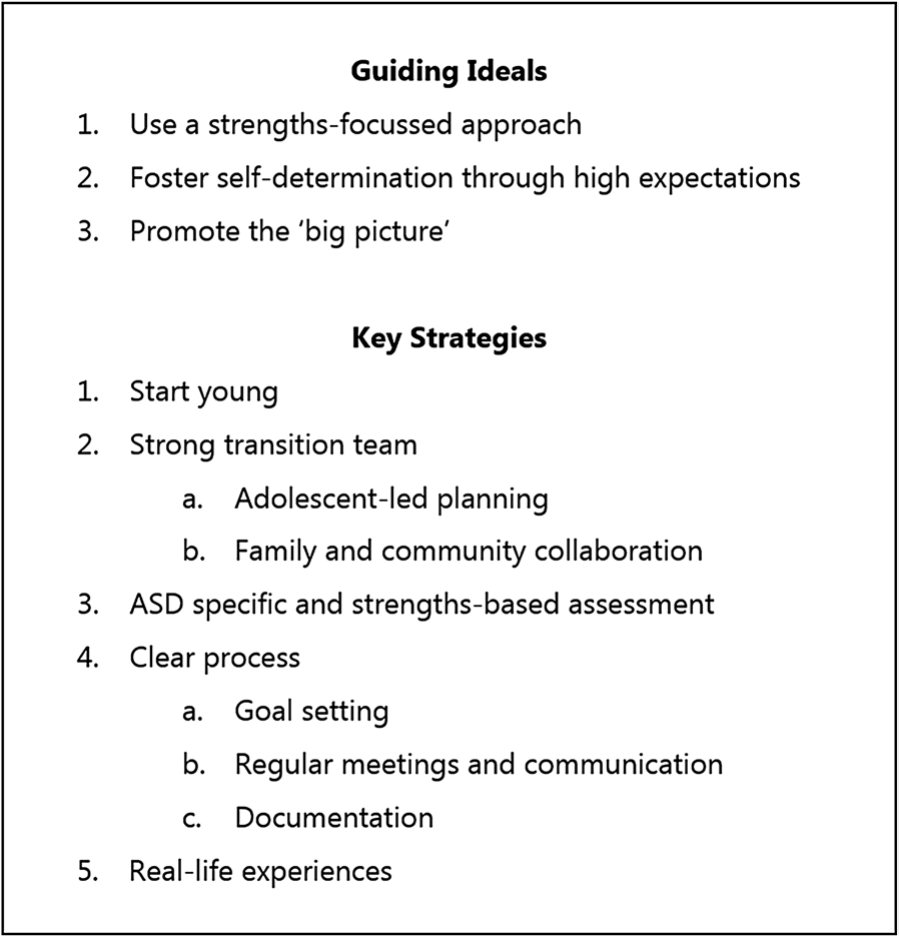
Transition planning objectives for adolescents on the autism spectrum

The findings of the literature review and the needs assessment shaped the transition planning objectives for adolescents on the autism spectrum (Fig. 1). The objectives consisted of three guiding ideals and five strategies to direct the overall development of the BOOST-A™. The ideal of ‘Promote the big picture’ is particularly important for adolescents on the autism spectrum, as it advocates the importance of assisting adolescents to understand what life will be like after school. Adolescents with autism may not implicitly understand the ‘big picture’ due to difficulties with abstract thought [41], which may cause them to less motivated to take part in transition planning. Therefore, assisting adolescents on the autism spectrum to understand the ‘big picture’ may enhance their motivation and participation in transition planning. The full findings of the needs assessment are reported elsewhere [42].

### The intervention: BOOST-A™

Using the transition planning objectives for adolescents on the autism spectrum (Fig. 1), the primary researcher (MH) developed the BOOST-A™. The BOOST-A™ was written in plain language at a year five reading level. This reading level has been recommended as being appropriate to disseminate health communication materials to the community, including low-literacy readers [43]. Feedback was obtained by a community reference group comprised of young people on the autism spectrum, parents and professionals throughout the development of the BOOST-A™ to ensure it met the needs of adolescents on the autism spectrum.

The BOOST-A™ is delivered in four online modules with an introduction via a website that requires a login. Table 1 shows the objectives addressed in each module. Each of the BOOST-A™ modules contains interactive cartoon videos that explain to the adolescent the overall purpose of transition planning, as well as the aim of each module. This links back to the guiding ideal identified in the needs assessment of ‘promote the big picture’.

**Table 1:** BOOST-A™ overview

Module	Description	Who and where	Objectives addressed
Introduction	Information about the process and what to expect in the program, and why it is important to engage in transition planning from an early age	Adolescent and their parent/s at home	1, 2, 4
1. About Me	Six activities to identify the adolescent’s interests, strengths, work preferences, training goals, life skills and learning style	Adolescent and their parent/s at home	2, 3, 4, 5
2. My Team	Guides how to identify a team of people to support the adolescent in their transition planning, and how to book the first team meeting. Adolescents choose how they want to get involved in the team meetings, with graded prompts provided	Adolescent and their parent/s at home	2, 4, 5
3. First Meeting	Guides the first meeting; when the team are provided with recommendations for job areas and goals based on the adolescent’s strengths and evidence from the literature	Adolescent, their parent/s and their team at the first meeting	2, 3, 4, 5
4. My Progress	Guides the progress meetings; when the team review how the adolescent’s goals are progressing; and discuss positive learning experiences	Adolescent, their parent/s and their team at subsequent team meetings	2, 3, 4, 5

The first module is ‘About Me’, in which the adolescent completes a number of activities to identify their interests and strengths. The focus is on leveraging the adolescent’s strengths, rather than focusing on their weaknesses [32]. The second module is ‘My Team’, which assists the adolescent and their parents to identify people who may support them in their transition planning journey. Being actively involved in transition planning and having people who provide tangible assistance and encouragement is pivotal to promoting self-determination [44, 45]. Therefore, this module encourages and supports the adolescent to become an integral and active member of the team. The third module, ‘First Meeting’, guides the team to develop goals; providing recommendations for goals that are based on the adolescent’s strengths and best-practice recommendations from the research literature, such as the importance of engaging in real life experiences [9]. The fourth module, ‘My Progress’, is completed by the team at all subsequent team meetings to review how the adolescent’s goals are progressing. This module encourages the team to reflect on progress in a positive manner and to view all experiences as learning opportunities, rather than failures.

### Pilot studies

Two pilot studies were conducted to determine the feasibility of the BOOST-A™, and to provide formative and process feedback. The pilot studies were:

1. Pilot A: with adolescents on the autism spectrum, their parents, teachers and other professionals; and
2. Pilot B: with allied health professionals.

*Pilot A* consisted of adolescents on the autism spectrum (n = 6), their parents (n = 6) and the professionals who worked with them (n = 12); who were recruited using convenience sampling from a database of people who had registered their interest in  the research project. Participants were asked to use the BOOST-A™ along with their team and to provide feedback on the process. All participants rated the BOOST-A™ as helpful, realistic and relevant (100%). Participants rated the ‘My Team’ section as low for usability (50%), and provided recommendations for improvement.

*Pilot B* included 88 allied health professionals, including speech pathologists (n = 26), psychologists (n = 29) and occupational therapists (n = 29) registered to practice in Australia, and recruited through allied health forums and professional networks. Participants completed an online survey comprised of questions about whether the BOOST-A™ was helpful, realistic, meaningful, relevant, and clear. Approximately three out of four (76%) of the allied health professionals rated the BOOST-A™ as appropriate, usable, and feasible; and 84% reported they would use BOOST-A™ in the future. Participants identified three main areas for improvement: (i) verbose language, (ii) need for support from parents in the ‘About Me’ section, and (iii) need for guidance overall in the program; and provided suggestions for improvement.

Based on feedback from both pilots, the BOOST-A™ was modified to enhance usability of the program, with the conversion from a Java platform to a web-based program that allowed for improved navigation and increased use of graphics and animations, and an overall reduction to the length of the program. The full results from these pilot studies are reported elsewhere [46].

### Trial design and procedures

**Fig. 2:**
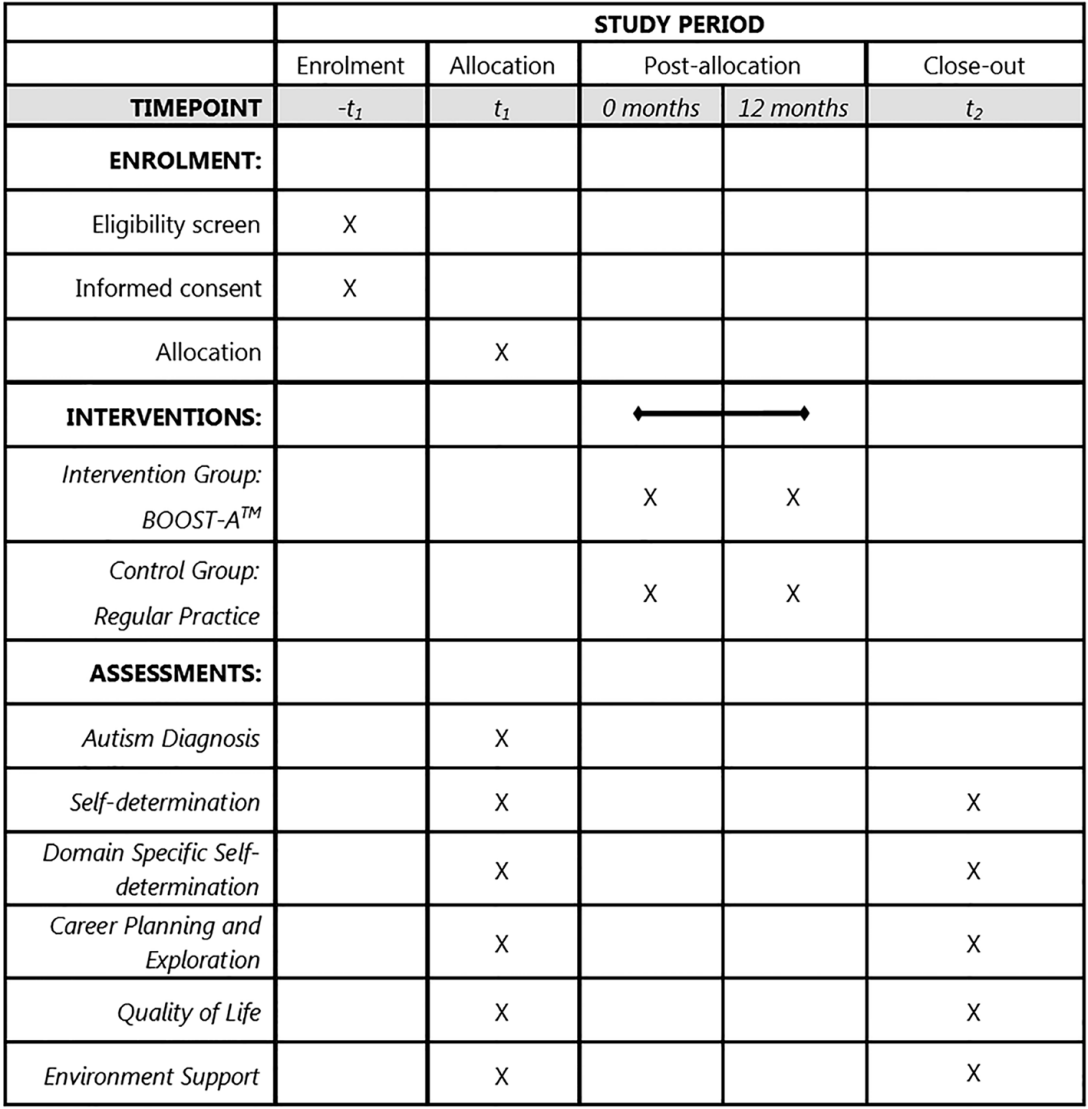
Schedule of enrolment, intervention and assessments

A controlled clinical trial [47] will be used to determine the effectiveness of the BOOST-A™ in improving the self-determination of adolescents on the autism spectrum; and in improving their outcomes of quality of life, access to environmental supports, and career planning skills. The trial will be a cluster group, two-arm, superiority trial with 1:1 allocation ratio. The trial will aim to detect any difference in these outcomes between participants in the intervention group (BOOST-A™) and a control group. Figure 2 shows the schedule of enrolment, intervention, and assessment for the trial. Participants in the intervention group will complete the BOOST-A™ at home and/or at school. Participants will complete the BOOST-A™ over a period of 12 months. This timeframe was chosen to ensure the participants have adequate time to complete all four modules, including the initial team meeting and at least one review meeting. Adherence will be monitored via website analytics, including number of modules completed and number of logins to the BOOST-A™ website. Participants allocated to the control group will participate in the existing post-school planning process used at their school (regular practice).

#### Participants

##### Identification and recruitment

Potential participants will be recruited via social media and community organization websites, flyers, and posters located in services for people on the autism spectrum. Community organizations, health professionals, and schools will be asked to email any potential participants directly. Recruitment material will consist of a flyer outlining the inclusion criteria and requirements of the trial. The flyer will request prospective participants to contact the primary researcher (MH) directly via email or telephone to register their interest in the trial. At the initial contact, each potential participant will be screened by MH for eligibility, and they will be sent the electronic participant information form and a link to an online consent form.

##### Inclusion and exclusion criteria

Inclusion criteria for participating in this trial are as follows:

- Adolescents diagnosed with Autism spectrum disorder, as defined by the diagnostic and statistical manual of mental disorders, fifth edition (DSM-5) [1] or the fourth edition (DSM-4) [3];
- Living in Australia;
- Able to read and write in English at a year five reading level;
- Enrolled in years 8–11 at school (including mainstream, special education or home-schooling programs); and
- Possess basic computer skills to enable use of the online BOOST-A™.

Adolescents will have a formal diagnosis of autism prior to participating in this trial. Diagnosis will be verified by the Social Responsiveness Scale-Second Edition (SRS-2) [48]. The SRS-2 is a diagnostic screening tool, and has been used in previous trials to verify diagnosis [49, 50]. Whilst it would be preferable to have used the autism diagnostic observation schedule (ADOS) [51] for diagnosis, it is not possible for the researchers to administer this 60 min assessment in person given the wide geographic distribution of participants across Australia. Further verification of diagnosis will be provided by parent report, as previous studies have verified the validity of diagnostic information reported by parents [52]. Exclusion criteria will be if the adolescent on the autism spectrum has an ID, as this will limit their ability to use the program, or if the student is enrolled in another transition planning program. Whilst it would have been ideal to complete an assessment of cognitive functioning for each participant, this is not feasible as the sample will be recruited from across Australia and the trial will be completed only online.

##### Treatment allocation

Following initial screening, potential participants will be allocated to either the intervention or control group according to the order in which they register interest. The first participant will be allocated to a group based on a coin toss completed by a researcher who will not be directly involved in liaising with participants. The exception to this allocation scheme is that if a newly enrolled participant is attending a school that a previously or currently enrolled trial participant attends, then the newly enrolled participant will be allocated to the same treatment group as the previous student. The reason for this is to reduce the risk of contamination, since school staff will be involved in administering the intervention. Participants will be blinded to their treatment; however, non-blinded allocation and lack of randomization could introduce potential bias. Once allocated to a group, participants will be sent the participant information and consent form. Forms for the intervention group will differ slightly from those for the control group, as they will contain information about the BOOST-A™, to ensure blinding to treatment is maintained. Participants who provide written consent will then be assigned a unique participant identification number. A strength of the trial is that the BOOST-A™ will be administered by parents and professionals who are not a part of the research team, thereby minimizing researcher bias. In addition, the primary researcher will have minimal information about the participants at time of randomization.

##### Sample size

Altman’s nomogram equation was used to determine the sample size. A total sample of n = 80 (n = 40 in each group) would be the minimum required to identify a standardized difference of 0.6 (i.e., Cohen’s *d*) [53], with a power of 80% and a critical alpha value of .05.

#### Data collection

Outcome data will be collected via an online survey using Qualtrics software (Version 2016). The survey containing the outcome measures for the trial will be emailed to participants at two data collection points: at baseline (Time point 1), and 12 months later (Time point 2). Participants will complete the online survey in their own environments, which could be home, work, or school. Demographic information will be collected from parents at Time point 1. This information will include the adolescent’s age, gender, year level at school, and their residential postcode (to determine socioeconomic status). It is anticipated that completing the online outcome measures will take 30–45 min each time. Participants will be given a leeway of two months after each scheduled data collection point to complete the outcome measures. Participants will be reminded, as needed, by the primary researcher via telephone and email to complete the outcome measures. The trial commenced on 26 November 2015 (Time Point 1), and data collection for the post-measures began on the 26 November 2016 (Time Point 2).

The Social Responsiveness Scale-Second Edition (SRS-2) [48] will be used to classify autism severity. Parents will complete the School Age Rating Form, which is designed for children aged 4–18 years. The SRS-2 consists of 65 items and can be administered in 15–20 min. The scale results in a total score and a *t*-*score*, which can be used as an index of severity of social deficits on the autism spectrum. Scores can be interpreted as falling into one of the following four categories: within normal limits, mild deficit, moderate deficit, and severe deficit. The scale has been standardized using a nationally representative sample, and has strong psychometric properties including high internal consistency (α = .95); construct validity (two strong factors); test–retest reliability (*r* = .88–.98); and interrater reliability (*r* = .91 between mothers and fathers) [48]. Studies have shown that the SRS-2 can detect clinically meaningful and statistically significant differences between typically developing children and those diagnosed with autism [54]. Additional independent variables for this trial will be comorbidities (including mental health), gender, age, and socioeconomic status.

#### Outcome measures

Outcome measures were determined based on a literature review of all suitable measures and their psychometric properties. Outcome measures were chosen based on the transition planning objectives for adolescents with autism, as identified in the needs assessment (Fig. 1). For example, fostering self-determination through high expectations is linked to the outcome of self-determination, and having a strong transition team is linked to measuring learning climate. As the BOOST-A™ was developed based on these objectives, the aim was to determine if it was effective in bringing about change in these areas. Particular emphasis was placed on each measure’s sensitivity to detect change. The outcome measures are all self-report, eliminating the risk of assessor bias. All of the outcome measures were trialed in Pilot A with six adolescents on the autism spectrum and their parents to ensure they were appropriate for use with these groups. Modifications were required for two of the questionnaires, as described below.

##### Primary outcome measure

The adolescent’s self-determination will be measured by the AIR Self-Determination Scale (AIR) [55], including their ability, knowledge and perceptions about their self-determination, and what opportunities exist for them to use their knowledge and abilities at home and school. Self-determination has been chosen as the primary outcome measures, as high self-determination is correlated with successful transition to employment in adolescents on the spectrum [25, 27–30]. The AIR consists of 24 items, as well as some free-form, short-answer questions. The AIR has good test–retest reliability (*r* = .74 based on two administrations three months apart), internal consistency (split half test *r* = .95), and construct validity (four factors explained 47% of the variance) [55]. Sensitivity to change was demonstrated in previous studies that used the AIR as an outcome measure for students with disabilities [56, 57]. The AIR has been established as a reliable instrument to use with adolescents on the autism spectrum [58].

##### Secondary outcome measures

Career planning and exploration will be measured by the Career Development Inventory—Australia (CDI-A) [60]. Career planning and exploration is defined as the ability to explore one’s skills and interests in relation to work, and to seek information related to one’s career to assist in making an informed decision [59]. The first two sections of the CDI-A [60] will be used for this trial as they specifically target career planning and exploration. These two sections contain 18 items and are valid and reliable, independent from the entire CDI-A [60]. The CDI-Australia has been found to have adequate internal consistency (career planning α = .84; career exploration α = .63), concurrent validity (*r* = .6–.8), and construct validity (four factors explaining 44.7% of the variance) [61].

Quality of life will be measured by the Personal Wellbeing Index-School Children (PWI-SC) [62], which is based on the Subjective Wellbeing Homeostasis Theory [63], which asserts that an individual operates to maintain their wellbeing around an average point. The PWI-SC contains seven items; one for each of the seven domains: standard of living, personal health, achievement in life, personal relationships, personal safety, community-connectedness, and future security [62]. The PWI-SC has high internal consistency (α = .82) and construct validity (comparative fit index = .96) [64]. Sensitivity to change was demonstrated in a study that used the PWI-SC as an outcome measure for a youth support program [65].

Environment support will be measured using the Learning Climate Questionnaire (LCQ) [66], which measures an individual’s perception of support from their team, or the environmental aspects that contribute to the development of self-determination. The LCQ consists of 15 items, and has been found to have good construct validity (one factor explaining 63% of the variance) and high internal consistency (α = .96) [66]. The LCQ has been used to evaluate how instructor’s support impacts on students’ learning in college, demonstrating its sensitivity to change [67]. Based on feedback from Pilot A, the LCQ was adapted to meet the needs of adolescents on the autism spectrum and to ensure it was transition planning specific by removing three questions and slightly modifying the language.

Domain specific self-determination was measured by the Transition Planning Objectives Scale (TPOS). The TPOS was designed specifically for this trial, because the authors could not identify an existing standardized tool that comprehensively evaluated the transition planning objectives identified in the needs assessment (Fig. 1). The primary researcher (MH) developed the scale based on the transition planning objectives and the measure of processes of care (MPOC) [68]. The MPOC was designed to assess parents’ perceptions of the care provided to their children by health professions in rehabilitation centers. Its underlying concepts align with those of the transition planning objectives, including enabling partnerships and family-centered care. The TPOS consists of 16 items, each of which addresses an objective in Fig. 1. Each item is rated on a 10-point Likert scale, anchored by strongly disagree to strongly agree. The measure was reviewed by the research team and then piloted with six adolescents on the autism spectrum and their parents, after which minor modifications were made. Since the validity and reliability of the transition planning objectives scale is not yet known, the data obtained from this measure will be interpreted and reported with caution. In addition, it is recommended that future studies aim to validate this outcome measure.

##### Statistical analysis

Simple descriptive statistics (frequencies and percentages for categorical variables; means, standard deviations, ranges for continuous variables) will be used to summarize the demographic and baseline profiles of participants. These baseline variables will include the assessment of autism severity (using the SRS-2), and the outcome measures described above. The Chi square or *t* test (as appropriate) will be used to compare the profiles of participants between the intervention and control groups. If any continuous data are found to be not normally distributed (from the Kolmogorov–Smirnov test), these data may either be transformed to improve their normality or analyzed using an appropriate non-parametric test.

Effectiveness of the intervention for the AIR, the primary outcome, will be determined by calculating the change from Time point 1 to Time point 2, and comparing the changes within and between intervention and control groups using dependent and independent *t* tests (or non-parametric Wilcoxon signed rank tests and Mann–Whitney U test if the data are not normally distributed). If the analysis reveals differences in baseline characteristics between the intervention and control groups, the analyses will be adjusted for these differences using a general linear model (GLM). Analysis of secondary outcomes will be performed using a multivariate ANOVA (MANOVA). Analyses will be performed using an intention-to-treat strategy, where participants will be classified as belonging to the group (intervention or control) to which they were initially allocated, regardless of the treatment they actually received. Participants who do not provide outcome data at Time point 2 will still be included in the trial, and their Time point 1 data will be used for Time point 2 to allow for intention-to-treat analysis of the data. The Statistical Package for the Social Sciences (SPSS v.22) will be used to analyze the data, and a p value of .05 will be used as the level of statistical significance in all inferential analyses.

#### Process evaluation

The process evaluation will explore the usability and feasibility of the BOOST-A™ to determine whether the results of the trial were influenced by external factors, such as the implementation process or contextual issues. The objectives of the process evaluation are to describe the participants’ experiences when using the BOOST-A™; participants’ perceptions of the usability of the BOOST-A™; and to identify facilitators and barriers impacting participants’ use of the BOOST-A™.

The process evaluation will use quantitative and qualitative feedback from participants in the intervention group [69]. Quantitative data will be obtained from website analytics, including the number of modules completed and the number of logins to the BOOST-A™ website. In addition, participants will complete a survey at the conclusion of the trial to provide feedback on the strengths of the program, suggestions for improvement, and the number of team meetings held during the trial period. Qualitative data will be collected using semi-structured interviews, to obtain in-depth information about participants’ experiences when using the BOOST-A™. Interviews will be conducted over the telephone with parents and adolescents together, within one month after completion of the trial. Interviews will be conducted by an independent researcher who has not been involved in previous stages of the project, to minimize any potential bias. Data collection will conclude when saturation is reached, or when interviews cease to provide any further insight into the topic of exploration [70]. Interviews will be audio-recorded, transcribed verbatim, and data de-identified. Transcripts will be analyzed using thematic analysis with constant comparison of the data within and across participants. In addition, the primary researcher (MH) will keep field notes for the duration of the trial, to document any incidental feedback obtained from participants, as well as any preconceptions she might have regarding the participants and/or their outcomes.

### Ethics, consent and permissions

Ethics approval to conduct this trial has been obtained from Curtin University Human Research Ethics Committee (approval number HR110/2014), and the Catholic Education Offices and Departments of Education in Western Australia, Victoria, Queensland, New South Wales, Tasmania and South Australia. Written informed consent will be obtained from all adult participants. Participants under 18 years of age will provide informed written assent, and their parents will provide informed written consent for their participation. Principals of the schools attended by participants in the intervention group will provide informed written approval for their staff to use the BOOST-A™; consent is not required by this group because the teachers are not taking part in data collection. Participants in the control group will be offered the opportunity to use the BOOST-A™ once the trial is complete, if it is proven to be effective in achieving the trial objectives.

The trial design and procedures will adhere to the National Statement on Ethical Conduct in Human Research [71] and the Australian Code for the Responsible Conduct of Research [72]. The trial is registered with the Australia and New Zealand Clinical Trial Registry (#ACTRN12615000119594). The trial was developed according to the Consolidated Standards of Reporting Trials (CONSORT) 2010 guidelines [73].

## Discussion

The BOOST-A™ is one of the first transition planning program that specifically targets and addresses the needs of adolescents on the autism spectrum. The needs assessment conducted prior to this trial revealed a number of unique areas of need that are specific to adolescents on the autism spectrum. For example, due to difficulties in gestalt processing [74] and abstract thinking, adolescents on the autism spectrum benefit from support to understand the ‘big picture’, and why they need to get a job after school. These areas have not been addressed in existing transition planning programs.

The BOOST-A™ will be one of the first transition planning programs to be empirically tested to provide evidence of its efficacy. The BOOST-A™ has been developed using a rigorous approach and by applying the PRECEDE-PROCEED model. The development phase involved a literature review and needs assessment, and the identification of transition planning objectives. Two pilot studies were completed to ensure the viability and feasibility of the program. The planned trial will determine the efficacy and usability of the BOOST-A™. To our knowledge, this level of rigor has not been applied to any existing transition planning interventions. In addition, the BOOST-A™, to the authors’ knowledge, is one of the first transition planning programs that is online. Having an online program may be beneficial for several reasons: increasing engagement of adolescents; allowing increased accessibility of the program from rural and remote areas; and allowing participants to use the intervention in their own homes, and at their own pace.

This trial will, to the authors’ knowledge, be the first national Australian research project of its kind to comprehensively address transition planning for adolescents on the autism spectrum. The objectives of the BOOST-A™ are in line with the major Australian Federal Government priority of increased workforce participation for Australians with disability, as outlined in the National Disability Strategy 2010–2020 [75]. Positive findings from this trial will have significant benefits for adolescents on the autism spectrum because the BOOST-A™ can be used to support them to find suitable employment as they move into adulthood. Participation in work is important for a number of reasons, including providing financial independence, and opportunities to develop social networks and supports [76]. It also provides a sense of identity, meaning, and purpose to people’s lives. Studies indicate that employed people on the autism spectrum experience meaningful improvements in quality of life [8]. Therefore, the BOOST-A™ may be able to support people on the autism spectrum to plan their pathway towards employment; an outcome that may ultimately enhance their quality of life and assist in reducing the unemployment of people with autism in Australia.

## Conclusions

The BOOST-A™ is the first online autism-specific transition planning program of its kind. This trial aims to provide evidence of the effectiveness of the BOOST-A™ to assist adolescents on the autism spectrum to successfully plan their transition from school into further study, training, or work.

## Abbreviations

BOOST-A: Better OutcOmes & Successful Transitions for Autism (BOOST-A)
ID: intellectual disability
